# Microbiological dataset of rural drinking water supplies in Zahedan, Iran

**DOI:** 10.1016/j.dib.2018.08.049

**Published:** 2018-08-23

**Authors:** Majid RadFard, Hamed Biglari, Hamed Soleimani, Hesam Akbari, Hamed Akbari, Hossein Faraji, Omid Dehghan, Abbas Abbasnia, Mona Hosseini, Amir Adibzadeh

**Affiliations:** a Health Research Center, Lifestyle Institute, Baqiyatallah University of Medical Sciences, Tehran, Iran; b Students Research Committee, Hamadan University of Medical Sciences, Hamadan, Iran; c Department of Medical Entomology and Vector Control, School of Public Health, Tehran University of Medical Sciences, Tehran, Iran; d Department of Environmental Health, School of public Health, Tehran University of Medical Sciences, Tehran, Iran; e Department of Environmental Health Engineering, School of Public Health, Gonabad University of Medical Sciences, Gonabad, Iran; f Department of Environmental Health Engineering, School of Health, Isfahan University of Medical Sciences, Isfahan, Iran

**Keywords:** Drinking water, Coliform and fecal coliform, Residual chlorine, HPC, Zahedan

## Abstract

The residual chlorine and microbial quality of drinking water in the Zahedan villages by a number of1221 samples from all 168 villages were collected between 2014–2015. Then the samples were evaluated using 9-tube fermentation methods and portable chlorine method test. Based on the microbial coliform and fecal coliform indices, the data indicated that the maximum and minimum controlling of the bacteria in the distribution network were in the winter (90.62%) and autumn (85.56%), respectively. Also in the reservoirs, the maximum and minimum controlling of the bacteria were in winter (93.49%) and autumn (87.35%), respectively. The residual chlorine was prepared in almost all of seasons.

**Specifications Table**

**Table:** 

Subject area	Water microbiology
More specific subject area	Microbiology
Type of data	Tables, Figure
How data was acquired	Data was collected between 2014–2015, 1221 microbial samples were prepared from the water facilities installed in these villages, and the microbial test was performed by MPN, P-A or MF methods and turbidity, temperature, and HPC tests based on the standard method.
Data format	Raw, Analyzed
Experimental factors	The mentioned parameters were analyzed according to the standards for water and wastewater treatment handbook.
Experimental features	The levels *of* microbial parameters were determined.
Data source location	Zahedan, Sistan and Baluchistan province, Iran
Data accessibility	The data are available whit this article
Related research article	Yousefi et al. [5]

**Value of the data**

• The water microbial controlling is very important for prepared the safe drinking water.
• The data are shown that the microbial water quality commonly prepared in Zahedan, Iran and they have consumed safe water.
• The reason for the higher percentage of desirable chlorine in the city of Zahedan in summer is that in the warm seasons, up to 1 mg/l of primary chlorine is due to the prevention of the prevalence of waterborne diseases.
• The data are indicated that the operator must more considerate to chlorination of drinking water in autumn season rather than other seasons.
• Is the data recommended to the reservoir and distribution of drinking water system need to be improving in the chlorination time.

## 1 Data

The residual chlorine and microbial quality of drinking water in the Zahedan villages by a number of1221 samples from all 168 villages were collected (Table 1) .Table 2 shows that the The Chlorometric data of drinking water resources of Zahedan villages, Table 3 shows that The Turbidity data in drinking water sources of Zahedan villages. Data indicated that the maximum and minimum controlling of the bacteria in the distribution network were in the winter and autumn respectively (Table 4). Also in the reservoirs, the maximum and minimum controlling of the bacteria were in winter and autumn respectively (Table 4). And Table 5 shows that the data of HPC microbial population count in reservoirs of Zahedan villages, Table 6 Comparison of desirable microbial index and free chlorine.

**Table 1:** The microbial data of drinking water resources of Zahedan villages.

Season	Number of	Number of	Number of	Number of	Number of	Number of
	villages covered	Turbidity tests	chlorometric tests	microbial tests	HPC tests	temperature tests
Spring	168	320	36,207	296	75	320
Summer	168	370	37,046	339	90	370
Fall	168	340	36,870	303	90	340
Winter	168	310	36,440	283	100	310

**Table 2:** The Chlorometric data of drinking water resources of Zahedan villages.

Season	Total number of chlorometric tests	Desirable percentage
Spring	36,207	97.4
Summer	37,046	92
Fall	36,870	96.68
Winter	36,440	99.29
Total	146,563	96.34

**Table 3:** The Turbidity data in drinking water sources of Zahedan villages.

Season	Total number	>1NTU	1-5,NTU	5<NTU	Desirable percentage	Desirable percentage
	of tests				from a health perspective	from an aesthetic
Spring	320	280	32	8	87.5	97.5
Summer	370	317	41	12	85.6	96.75
Fall	340	290	36	14	85.27	95.5
Winter	310	275	29	6	88.7	98
Total	1340	1162	138	40	–	–

**Table 4:** The microbial data of distribution network of Zahedan villages.

	Microbiological test of network					Microbiological test of reservoirs				
**Season**	Total	Clean	Coliform	Fecal	Desirable	Total	Clean	Coliform	Fecal	Desirable
	number	number		coliform	percentage	number	number		coliform	percentage
Spring	204	180	21	3	88.23	92	84	6	2	91.3
Summer	236	206	27	3	87.28	103	93	8	2	90.29
Fall	216	185	26	5	85.56	87	76	8	3	87.35
Winter	160	145	13	2	90.62	123	115	7	1	93.49
Total	816	716	87	13	–	405	368	29	8	–

**Table 5:** The data of HPC microbial population count in reservoirs of Zahedan villages.

Season	Number	≤100	>100-≤200	>200-≤250	>250-≤500	>500	Mean	Minimum	Maximum
Spring	75	54	12	9	–	–	76.72	< 1	237
Summer	100	77	13	8	2	–	87.36	< 1	329
Fall	90	69	10	8	2	1	95.72	< 1	623
Winter	100	90	5	5	–	–	70.49	< 1	226

**Table 6:** Comparison of desirable microbial index and free chlorine.

Season	The desirability of chlorine	The desirability of microbial
Spring	99.24	98.31
Summer	99.45	98.52
Fall	99.4	97.35
Winter	99.31	98.93
Mean	99.35	98.27

## 2 Experimental design, materials and methods

### 2.1 Study area description

**Fig. 1:**
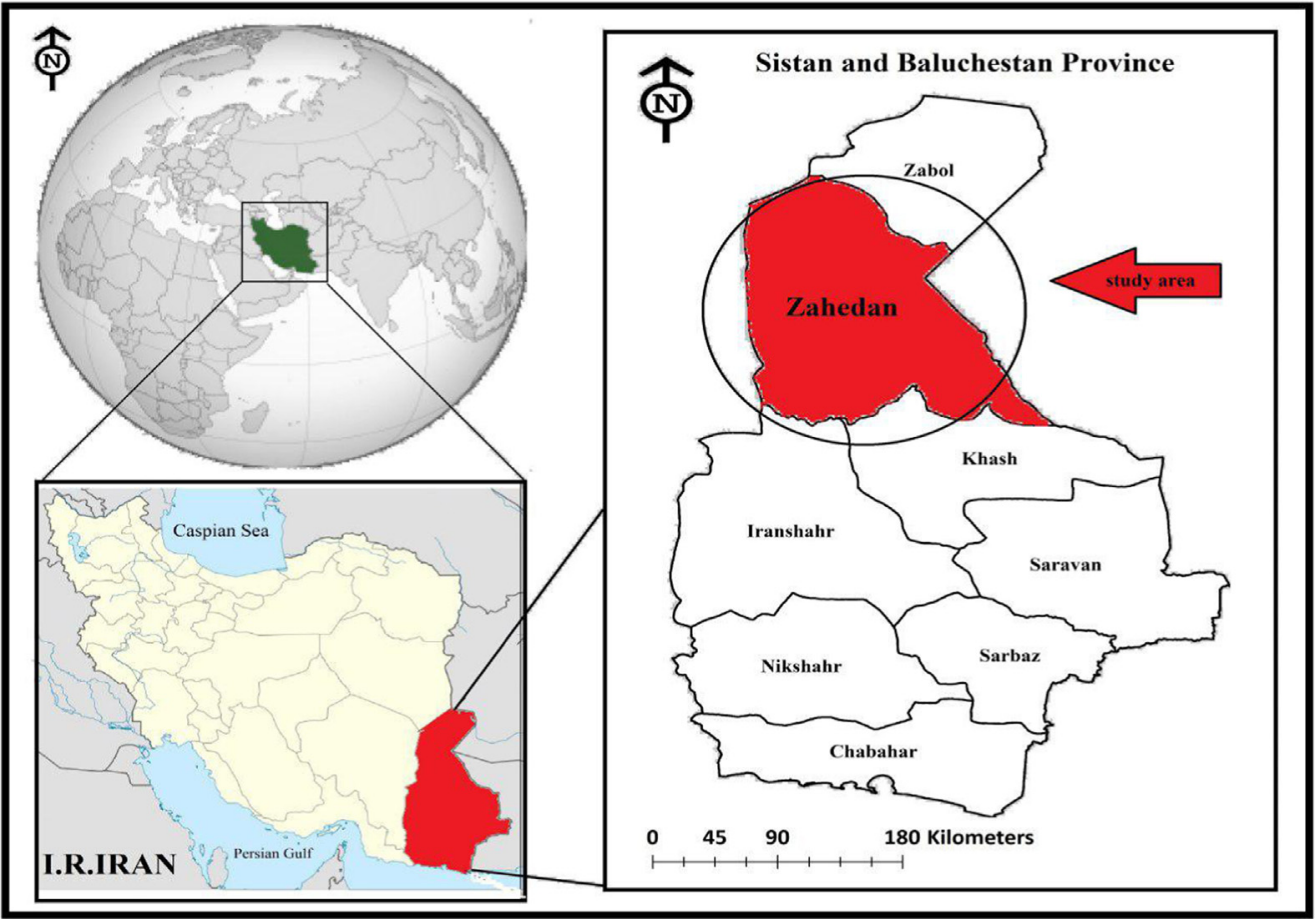
Geographical map of the site study.

Zahedan city is located in Sistan and Baluchistan province of Iran encompassing an area of about 55.7 km^2^ (Fig. 1) and its aquifers are located in South-East Iran between the latitudes 29° 30′ 45′′ N and longitudes 60° 51′ 25′′ E [1], [2]. The subjected study area is a semi-flat plain region with a gentle slope toward the south has a warm, temperate climate with an annual average of 18.3 °C in which the highest and lowest temperatures are 42.5 °C and −12.6 °C, respectively [3], [4].

### 2.2 Determination of microbial contamination in drinking water

In order to evaluate the microbial quality of drinking water in villages of Zahedan city, 168 villages were selected as a comprehensive sample of all villages in this city. Between 2014–2015, 1221 microbial samples were prepared from the water facilities installed in these villages, and the microbial test was performed by MPN, P-A or MF methods and turbidity, temperature, and HPC tests based on the standard method [5], [6], [7], [8], [9], [10], [11], [12], [13], [14], [15], [16], [17].
